# Case report: An elderly woman with recurrent syncope after pacemaker implantation

**DOI:** 10.3389/fcvm.2023.1117244

**Published:** 2023-03-08

**Authors:** Yanxiang Sun, Li Feng, Xuansheng Huang, Bing Hu, Yong Yuan

**Affiliations:** Department of Cardiology, Zhongshan City People's Hospital, Zhongshan, China

**Keywords:** permanent pacemaker implantation, loss of capture, pulmonary embolism, syncope, electrophysiology phenomenon

## Abstract

Syncope caused by atrioventricular block may occur as a result of a cardiac vasodepressor reflex. This article reports on a case of recurrent syncope in an 80-year-old woman with high-grade atrioventricular block, documented by electrocardiographic monitoring after pacemaker implantation. Pacemaker testing revealed stable impedance and sensing but a clear increase in the ventricular capture threshold at outputs. This case is unusual because the patient's primary diagnosis was non-cardiac. However, a combination of high D-dimer, hypoxemia, and computerized tomography scan of the pulmonary artery confirmed the diagnosis of pulmonary embolism (PE). With 1 month of anticoagulant therapy, the ventricular capture threshold gradually dropped to the normal range and syncope resolved. This is the first report of an electrophysiological phenomenon detected by pacemaker testing in a patient with syncope arising from PE.

## Introduction

The clinical picture of pulmonary embolism (PE) is variable, and it remains challenging to diagnose and a frequent cause of death. Close attention should be paid to the symptom of syncope, although data show that PE is identified in <1% of patients with syncope and in <3% of hospitalized patients with syncope (1, 2). Bradycardia-induced syncope during PE is rarely reported, and the underlying mechanism is not well-understood. Some studies suggest that PE can trigger the vasovagal reflex (3, 4), which leads to bradycardia. However, no studies have yet confirmed the changes in electrophysiology induced by the vagal response in PE, and investigation of the relationship between electrocardiographic (ECG) abnormalities and electrophysiology under the purview of pacemaker research has been limited. The purpose of the present case study was to further assess the relationship between acute PE and cardiac electrical signals.

## Case presentation

**Figure 1:**
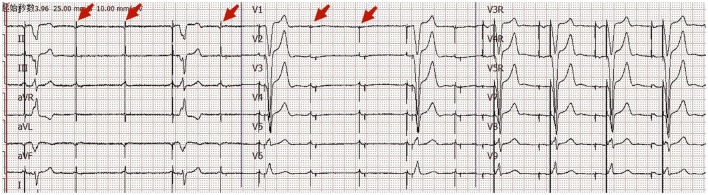
Eighteen-channel surface ECG. ECG on admission showed transient loss of ventricular pacing capture.

**Figure 2:**
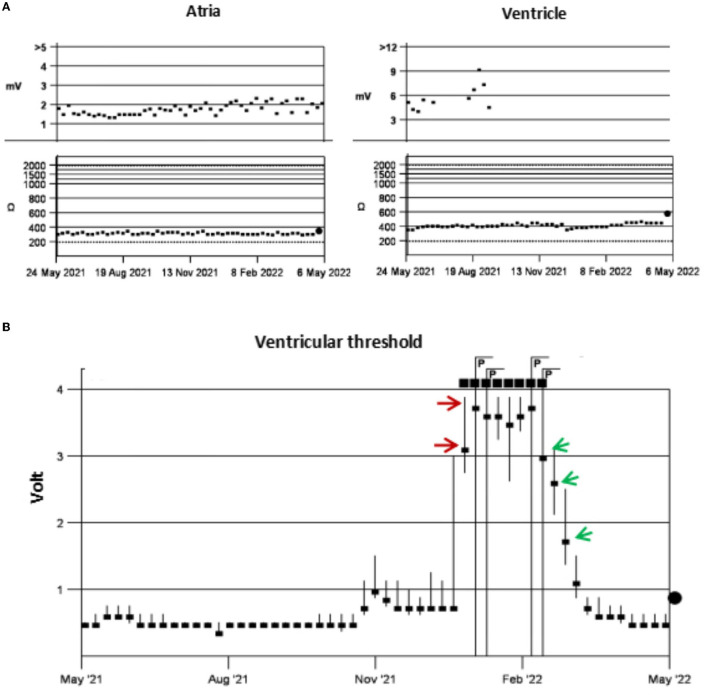
Pacing test. Testing showed stable impedance and sensing in the atria and ventricle, with no ventricular sensing data after October 2021 **(A)**. There was a clear increase in the ventricular capture threshold in early January 2022 (red arrows) and this gradually decreased to normal after 1 month of anticoagulant therapy (green arrows) **(B)**.

**Figure 3:**
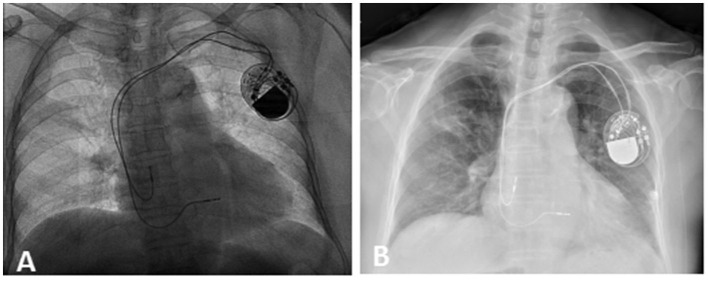
Chest x-ray. X-ray after the first pacemaker operation on September 12, 2019 **(A)** and on February 16, 2022 **(B)**. This showed correct lead position on admission.

A patient in her 80's with a history of pacemaker implantation presented with recurrent syncope for the past 2 months. She denied having chest pain, palpitations, or shortness of breath before the onset of syncope. She exhibited Mobitz II atrioventricular (AV) block and underwent a dual-chamber pacemaker implantation (St. Jude Medical Zephyr XL DR 5826) 3 years ago. She had a history of hypertension and diabetes for the past 5 years and was treated in the outpatient clinic. On initial evaluation, her blood pressure was 170/68 mmHg, heart rate 70 beats per minute, and oxygen saturation 92% on room air. Physical examination, including examination of the heart and lungs and a neurological examination, was normal. There were no recent changes in medication. She experienced recurrent syncope during the loss of ventricular pacing capture. ECG (Figure 1) showed a transient loss of ventricular pacing capture. At the last follow-up visit, pacing outputs were set at A: 2.0V, V: 2.2 V, with a programmed atrioventricular (AV) delay of 200 ms (DDD). Pacemaker testing revealed both stable impedance and stable sensing in the atria and ventricles (Figure 2A) and a normal atrial output threshold (0.5 to 0.75 volts under a 0.4-ms pulse width) but a clear increase in ventricular capture threshold at outputs 2 months previously (Figure 2B). The period of threshold elevation was consistent with the reported syncopal episodes. However, an anteroposterior chest x-ray showed correct atrial and ventricular pacing lead positions (Figures 3A, B). The patient had also undergone craniocerebral CT examination before admission, which revealed no brain-related diseases.

**Figure 4:**
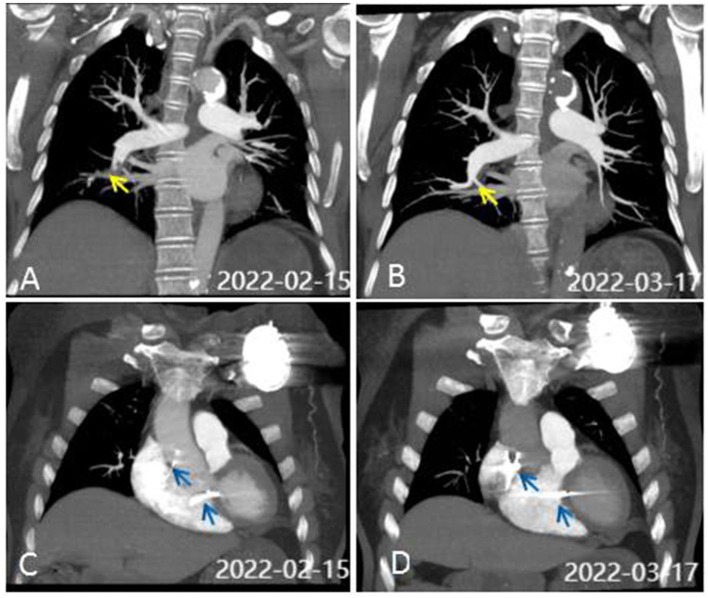
CT scan of the pulmonary artery. **(A)** CTA on admission showed pulmonary embolism (PE) in the right lower pulmonary arterial branch. Perfusion defects are identified by yellow arrows. **(B)** CTA was normal after 1 month of anticoagulant therapy. **(C, D)** show the positions of the RV and atrial leads (blue arrows) on PA view on admission **(C)** and 1 month later **(D)**.

What are the possible causes of the change in the ventricular capture threshold? We adjusted the pulse width and improved the output power of the pacemaker to ensure normal pacing. The results of initial testing showed normal cardiac enzymes and electrolytes but markedly high D-dimer levels (4.41 mg/L) and hypoxemia (PaO_2_ 62 mmHg). Therefore, a computerized tomography **(**CT) scan of the pulmonary artery was performed in view of the presenting symptoms; this showed PE in the right lower pulmonary arterial branch (Figure 4A). Further testing for hypercoagulable disorders, autoimmune diseases, tumors, and deep vein thrombosis revealed no abnormalities. An echocardiogram revealed ventricular septal hypertrophy (12 mm) and a mild elevation in pulmonary arterial systolic pressure (35 mmHg). Furthermore, a single-photon emission computed tomography (99m-Tc -pyrophosphate nuclear scintigraphy) was performed to rule out cardiomyopathy, and the results were normal. Subsequently, following treatment with anticoagulation and adjustments to the pacing output, the patient was discharged without symptoms after 10 days. After 1 month, the ventricular capture threshold gradually fell back to the normal range; a reexamination CT scan of the pulmonary artery indicated no evidence of PE (Figure 4B), and arterial oxygen pressure (PaO_2_ 82 mmHg) and D-dimer levels (0.2 mg/L) were normal. Figures 4C, D depict the positions of the right ventricular and atrial leads, respectively, from a posteroanterior view, at the time of admission and 1 month later. The patient was well for 10 months, with no evidence of any recurrence of syncope.

## Discussion

Bradycardia-induced syncope in cases of PE is rarely reported, and the mechanism underlying this phenomenon remains poorly understood. This report presents an unusual case of PE characterized by symptomatic bradycardia and transient loss of ventricular pacing capture. Despite the absence of chest pain or shortness of breath, the patient suffered from recurrent syncope due to bradycardia. In one series of angiographically confirmed cases of PE, it was discovered that 13% of the patients experienced an episode of syncope (5, 6). Of these, more than 80% had a massive embolism, defined as an obstruction of 50% of the pulmonary circulation. Acute right ventricular failure in these patients impairs left ventricular filling, and the ensuing hypotension causes syncope. However, in our case, the patient did not have a massive embolism, her blood pressure was normal, and there were no malignant arrhythmias. Furthermore, the patient had not started any new medications since late December 2021, making it unlikely that syncope was related to any of these factors.

The common causes of an increase in pacing threshold include acute myocardial ischemia, hypoxia, hyperkalemia, and electrode-related problems, such as electrode dislocation, perforation, and fracture. However, these causes were ruled out in this case. Large cohort clinical studies have shown that sinus bradycardia and first-degree AV block may occur in over 2 and 3.5% of patients with PE, respectively (7). Some studies suggest that PE induces bradycardia, as a result of triggering a vasovagal reflex (3, 4). Patients with PE are in a state of high adrenergic energy, which stimulates the mechanoreceptors in the inferior ventricle to reduce sympathetic tone and increase parasympathetic excitability. In addition, patients with PE have been found to exhibit conduction block due to neurohumoral mechanisms that regulate pulmonary artery pressure and increase ventricular pressure (8). Bradycardia after PE may occur if a defect is present in the sympathetic reflex or the right atrial pressure receptors or if their function is inadequate to compensate for the elevated parasympathetic activity (9, 10).

Furthermore, experimental data have shown that adenosine 5′-triphiosphate (ATP) could also play a role in bradycardia and syncope in a subset of patients with PE (11). The proposed mechanism involves the activation of platelets in the lungs, which leads to the localized release of ATP, followed by ATP-triggered pulmonary–pulmonary and cardio-cardiac vagal reflexes. The presence of bradycardia indicates that the block is caused by the influence of increased parasympathetic activity on the sinoatrial (SA) and atrioventricular (AV) nodes (12). Further animal studies are needed to confirm the potential role of PE in increasing the pacing threshold.

## Conclusion

PE presenting with bradycardia is rare and is difficult to diagnose. Therefore, physicians must be vigilant with patients who exhibit syncope, especially those with syncope who have an implanted pacemaker, as this symptom may be a “forgotten sign” of life-threatening PE. Missing a diagnosis of PE can increase patient morbidity and mortality. Early diagnosis and appropriate treatment are required to improve clinical outcomes in patients with PE. This case report highlights the correlation between PE and the activation of the parasympathetic nervous system, which raises the cardiac pacing threshold in the SA or VA node. The pacemaker parameters in this patient revealed this electrophysiological phenomenon, supporting previous hypotheses.

## Data availability statement

The original contributions presented in the study are included in the article/supplementary material, further inquiries can be directed to the corresponding authors.

## Ethics statement

Written informed consent was obtained from the individual(s) for the publication of any potentially identifiable images or data included in this article.

## Author contributions

YS, LF, XH, and BH: original draft preparation. YS and YY: review and editing. All authors have read and agreed to the published version of the manuscript.
